# Bicontinuous oxide heteroepitaxy with enhanced photoconductivity

**DOI:** 10.1038/s41467-022-35385-0

**Published:** 2023-01-03

**Authors:** Pao-Wen Shao, Yi-Xian Wu, Wei-Han Chen, Mojue Zhang, Minyi Dai, Yen-Chien Kuo, Shang-Hsien Hsieh, Yi-Cheng Tang, Po-Liang Liu, Pu Yu, Yuang Chen, Rong Huang, Chia-Hao Chen, Ju-Hung Hsu, Yi-Chun Chen, Jia-Mian Hu, Ying-Hao Chu

**Affiliations:** 1 Department of Materials Science and Engineering, National Yang Ming Chiao Tung University, Hsinchu, 30010 Taiwan; 2 Department of Materials Science and Engineering, University of Wisconsin-Madison, Madison, WI 53706 USA; 3 National Synchrotron Radiation Research Center, Hsinchu, 30076 Taiwan; 4 Graduate Institute of Precision Engineering, National Chung Hsing University, Taichung, 402 Taiwan; 5 State Key Laboratory of Low Dimensional Quantum Physics and Department of Physics, Tsinghua University, 100084 Beijing, People’s Republic of China; 6 Key Laboratory of Polar Materials and Devices, Ministry of Education, East China Normal University, 200241 Shanghai, China; 7 Integrated Service Technology, Hsinchu, Taiwan; 8 Department of Physics, National Cheng Kung University, Tainan, 70101 Taiwan; 9 Department of Materials Science and Engineering, National Tsing Hua University, Hsinchu, 30013 Taiwan

**Keywords:** Nanoscale materials, Materials for devices

## Abstract

Self-assembled systems have recently attracted extensive attention because they can display a wide range of phase morphologies in nanocomposites, providing a new arena to explore novel phenomena. Among these morphologies, a bicontinuous structure is highly desirable based on its high interface-to-volume ratio and 3D interconnectivity. A bicontinuous nickel oxide (NiO) and tin dioxide (SnO_2_) heteroepitaxial nanocomposite is revealed here. By controlling their concentration, we fabricated tuneable self-assembled nanostructures from pillars to bicontinuous structures, as evidenced by TEM-energy-dispersive X-ray spectroscopy with a tortuous compositional distribution. The experimentally observed growth modes are consistent with predictions by first-principles calculations. Phase-field simulations are performed to understand 3D microstructure formation and extract key thermodynamic parameters for predicting microstructure morphologies in SnO_2_:NiO nanocomposites of other concentrations. Furthermore, we demonstrate significantly enhanced photovoltaic properties in a bicontinuous SnO_2_:NiO nanocomposite macroscopically and microscopically. This research shows a pathway to developing innovative solar cell and photodetector devices based on self-assembled oxides.

## Abstract (web summary)

Self-assembled nanocomposites present opportunities for a range of phase morphologies and desirable properties. Here authors present tuneable self-assembled nanostructures in the SnO2:NiO system; the double-percolated system expands the design of self-assembled oxides for practical applications, e.g. in optoelectronics.

## Introduction

Composites outperforming the properties of constituents benefit from functional enhancement due to the addition of the second phase. Typically, desirable phases with designed dimensions and sizes are mixed in composite materials during the fabrication process. Thus, the acquisition of enhanced properties relies on the control of material distribution. This critical issue can be overcome via a thermodynamic approach based on the phase separation process. For example, the ideal microstructure for enhancing flux-pinning in high-temperature superconductors was to introduce non-superconducting columnar defects within the oxide superconductors. Goyal et al. employed phase separation and strain-driven self-assembly to introduce self-assembled, vertical nanocolumns of insulating BaZrO_3_ within YBCO, resulting in massive enhancement of flux-pinning^1,2^. The original attention was given to the additional phase to tune the functionality of the primary phase, such as the modification of magnetotransport behaviors of (La,Ca)MnO_3_ via the strain imposed by MgO^3^. Since then, this approach of phase-separation and strain-driven self-assembly of vertical nanocolumns of one material within another has been used in various oxide and nitride systems and has also been referred to as vertically aligned nanocomposites^4–11^. Because the strain states of the constituent phases remain independent of the thickness of the heterostructure, a research field based on this bottom-up methodology to obtain self-assembled vertical aligned nanocomposites (VANs) was formed to enhance the structural coupling^4^. An extensive exploration of strain-mediated systems has been carried out to acquire desired properties such as ferroelectricity and magnetic anisotropy^5^.

Later, the focus was placed on the coupling of two coexisting phases, resulting in enhanced studies on magnetoelectric nanocomposites^6,7^. However, for current structures, especially in self-assembled oxide heteroepitaxy, one critical characteristic in these systems is the existence of a network composed of two different phases, with one as the major continuous matrix and the other as the isolated nanostructure, representing a classic structure attributed to the mechanism of nucleation and growth^8^. However, few works have focused on checkerboard Mn-doped CoFe_2_O_4_^9^ and nanomaze (La,Sr)MnO_3_/ZnO heterostructures^10^. A bicontinuous heterostructure has only been observed in the isostructural system of TiO_2_ and VO_2_^11^. However, such a microstructure is commonly seen in alloy and polymer systems. In metallurgy, a bicontinuous double percolated morphology can be obtained via spinodal decomposition^12,13^, in which one thermodynamic phase spontaneously separates into two different phases. This differs from the traditional nucleation and growth process, where a nucleation barrier has to be overcome. In 2001, attention was given to nanoporosity after the selective dissolution of electrochemically active elements. The resulting nanoporous sponge was suitable for catalytic and electrochemical applications^12^. Moreover, in organic solar cells, the formation of bicontinuous phases, named bulk heterojunctions, significantly improves due to a dramatic increase in the interface area with strong charge interactions at the interface^14^. Thus, creating a bicontinuous heterostructure can enhance the charge interactions in oxide systems to boost the photovoltaic effects or photoconduction performance.

In this study, we propose a bottom-up approach to create a self-assembled oxide system with tuneable structural architectures (i.e., tortuous bicontinuous structure or isolated pillar structure) in epitaxial SnO_2_:NiO nanocomposites. The reason for selecting the SnO_2_:NiO system as the model system is (1) the structural similarity in rock salt NiO and rutile SnO_2_, in which the cations of both tin and nickel occupy octahedral sites. Moreover, they are both structurally compatible with sapphire substrates. (2) The SnO_2_:NiO system reveals excellent potential in device applications such as photodetectors^15^, gas sensors^16,17^, and photocatalysts^18^. In addition, the p/n junction in this system has been widely explored^19^. Therefore, the dominating interface characteristic of charge transport could be studied both macroscopically and microscopically.

## Results

**Fig. 1:**
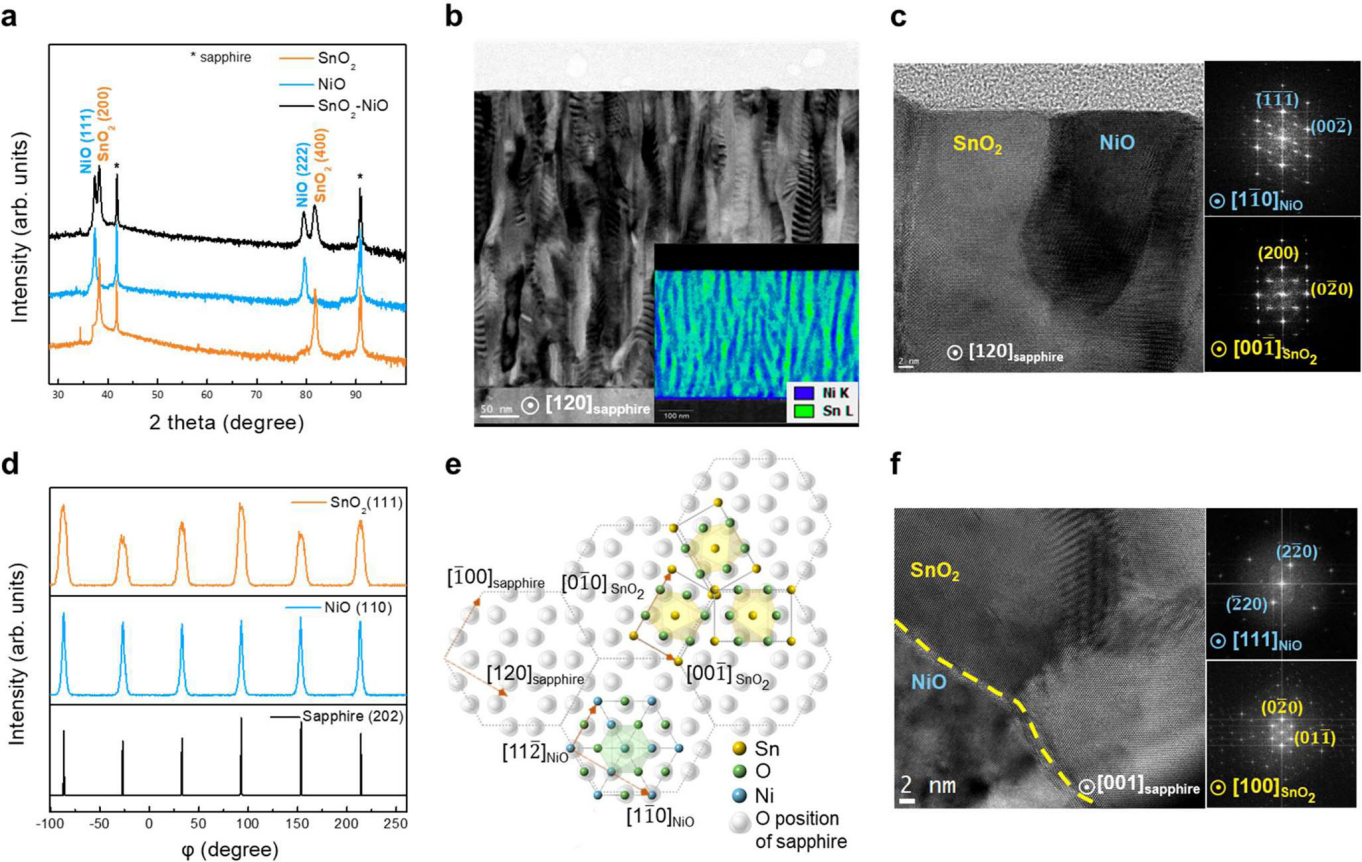
Bicontinuous nanostructures of SnO_2_:NiO/sapphire with a composition ratio of 1:1 (bicontinuous structure). **a** X-ray scans in theta−2theta mode of SnO_2_:NiO/sapphire. **b** Cross-sectional TEM along the *[120]*_*sapphire*_ zone axis. From top to bottom: surface, SnO_2_:NiO, and sapphire. The inset shows the SEM‒EDS elemental mapping at the same scale. The distribution of nickel and tin is combined in a layered image. **c** The larger scale of the cross-sectional HRTEM image along the *[120]*_*sapphire*_ zone axis. Inset: FFT of NiO and SnO_2_. **d** In-plane phi-scan patterns of sapphire (202) reflection, NiO (110) reflection, and SnO_2_ (111) reflection. **e** Lattice projection along the epitaxial axis (*[400]*_*SnO2*_||*[111]*_*NiO*_||*[001]*_*sapphire*_). The orange arrows represent the in-plane orientation relationship. The projected positions of oxygen octahedra in SnO_2_ and NiO overlap with that of sapphire. **f** Plane-view HRTEM image along the *[001]*_*sapphire*_ zone axis. The yellow dotted line displays the boundary between NiO and SnO_2_. Inset: FFT of NiO and SnO_2_.

The details on growth can be found in the methods section. Structural analysis was conducted by X-ray diffraction (XRD) and transmission electron microscopy (TEM) to examine sample quality and determine the epitaxial relationship. Figure 1a displays typical X-ray scans of samples of SnO_2_:NiO/sapphire, pure SnO_2_/sapphire, and NiO/sapphire in *θ*-2*θ* mode. Compared with the pure samples, the existence of both NiO(*lll*) and SnO_2_(*00l*) diffraction peaks presents a single out-of-plane orientation of SnO_2_:NiO/sapphire. Furthermore, X-ray rocking curves (Supplementary Fig. 1) display full-width at half-maximum (FWHM) values of 0.9° for NiO(111) and 1.2° for SnO_2_(200), delivering evidence of the superior crystallinity of the heterostructure. After the annealing process (the details on the annealing conditions can be found in the Methods section), the cross-sectional TEM image (Fig. 1b) displays a pillar-like percolated feature of SnO_2_:NiO/sapphire. STEM (Supplementary Fig. 2) and HRTEM images (Fig. 1c) further confirm that the termination of the NiO pillar is connected with SnO_2_ rather than the substrate, representing a bicontinuous feature of SnO_2_:NiO, which is further identified by X-ray energy-dispersive spectroscopy (EDS) with the compositional distribution of nickel and tin in the inset of Fig. 1b and Supplementary Fig. 2. Considering the disparate crystal structures of SnO_2_ (rutile) and NiO (rock salt), this result shows the feasibility of bicontinuous features for self-assembled nanocomposites. In addition, the epitaxial relationship (i.e., *[100]*_*SnO2*_||*[111]*_*NiO*_||*[001]*_*sapphire*_, and *[*$00\bar{1}$*]*_*SnO2*_||*[*$1\bar{1}0$*]*_*NiO*_||*[120]*_*sapphire*_) of the self-assembled heteroepitaxial film is further characterized by larger-scale HRTEM (Fig. 1c) and the fast Fourier transform (FFT) in the inset. In addition, the HRTEM of a near edge-on SnO_2_/NiO interface is not atomically sharp and flat (Supplementary Fig. 3), indicating that the tilt interface is beyond the epitaxial relationship, and bicontinuous features with various curved interfaces are expected.

Figure 1d reveals the XRD *φ*-scans of the SnO_2_(111), NiO(110), and sapphire(202) reflections. For sapphire and NiO, there are six reflection peaks at every 60° interval, which is consistent with the sixfold symmetry of both NiO (cubic with two domains) along *[111]*_*NiO*_ and sapphire (hexagonal) along the c-axis. Tetragonal SnO_2_ is expected to display two reflection peaks at 180° intervals owing to twofold symmetry along *[100]*_*SnO2*_. However, it reveals six peaks at the same *φ* angles as those of NiO and sapphire. This result indicates that SnO_2_ consists of three domains with a rotation of 120°, which are compatible with the sapphire substrate in Fig. 1e. Furthermore, for rutile SnO_2_ and rock salt NiO, the projected positions of oxygen in octahedrons could overlap with those of oxygen in sapphire. In contrast, the projected positions of tin and nickel could coincide with that of aluminum in sapphire (Supplementary Fig. 4). Therefore, the heteroepitaxy of SnO_2_:NiO/sapphire could be considered a continuous expansion of coherent hexagonal atomic arrangements, which is energetically favorable at the interface. Combined with the plane-view HRTEM and FFTs in Fig. 1f, the epitaxial relationship is further confirmed.

We further elaborate on the reason for forming SnO_2_ and NiO phases instead of trigonal NiSnO_3_. First, the fabrication temperature (i.e., 450–650 °C) for polycrystalline NiSnO_3_ is similar to that of SnO_2_ and NiO, ruling out inappropriate growth conditions^20^. Second, NiSnO_3_ displays trigonal crystal symmetry, yet the lattice misfit from the sapphire substrate is 2.6%^21^. Therefore, the projection position of oxygen in NiSnO_3_ is similar to that of oxygen in sapphire, suggesting that the NiSnO_3_ formation is not energetically favorable, likely due to the energy cost of the elastic deformation (in Supplementary Fig. 5).

**Fig. 2:**
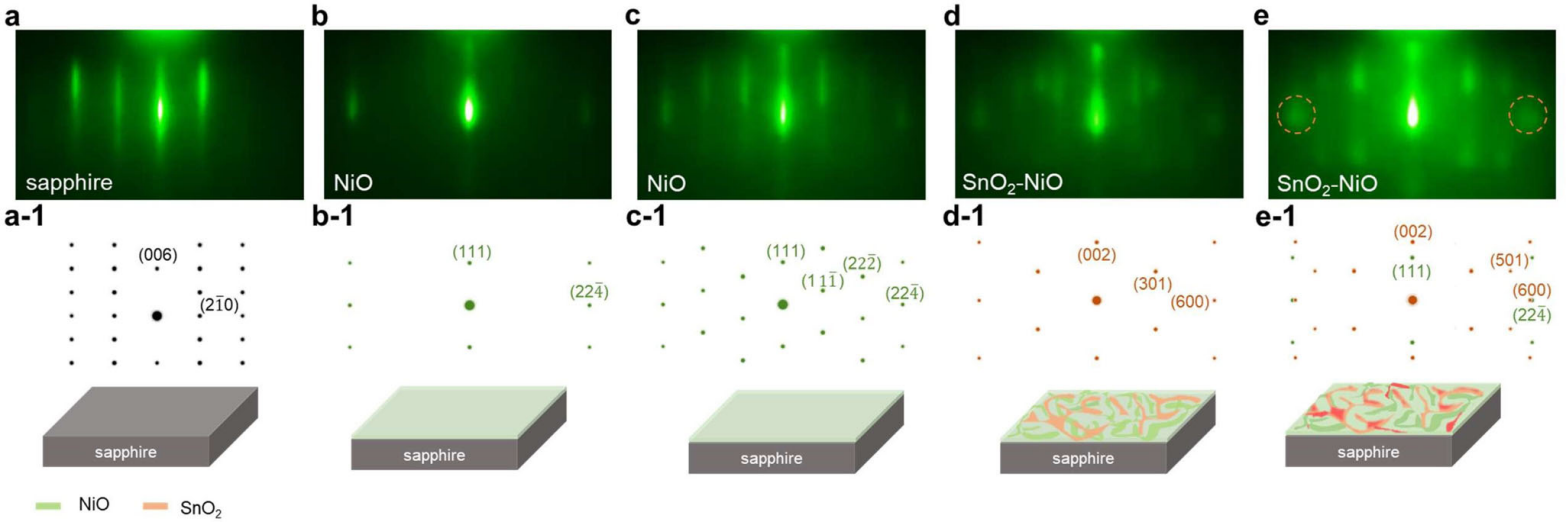
In situ RHEED patterns of the epitaxial growth of SnO_2_:NiO on c-plane sapphire along *[120]*_*sapphire*_ at 750 °C. **a** Initial state of c-plane sapphire. **a-1** The corresponding diffraction pattern of sapphire and schematics. **b**–**e** After 75, 125, 175, 225 pulses of deposition. **b1**–**e1** The corresponding diffraction patterns of NiO (green), SnO_2_:NiO (orange), and schematics.

**Fig. 3:**
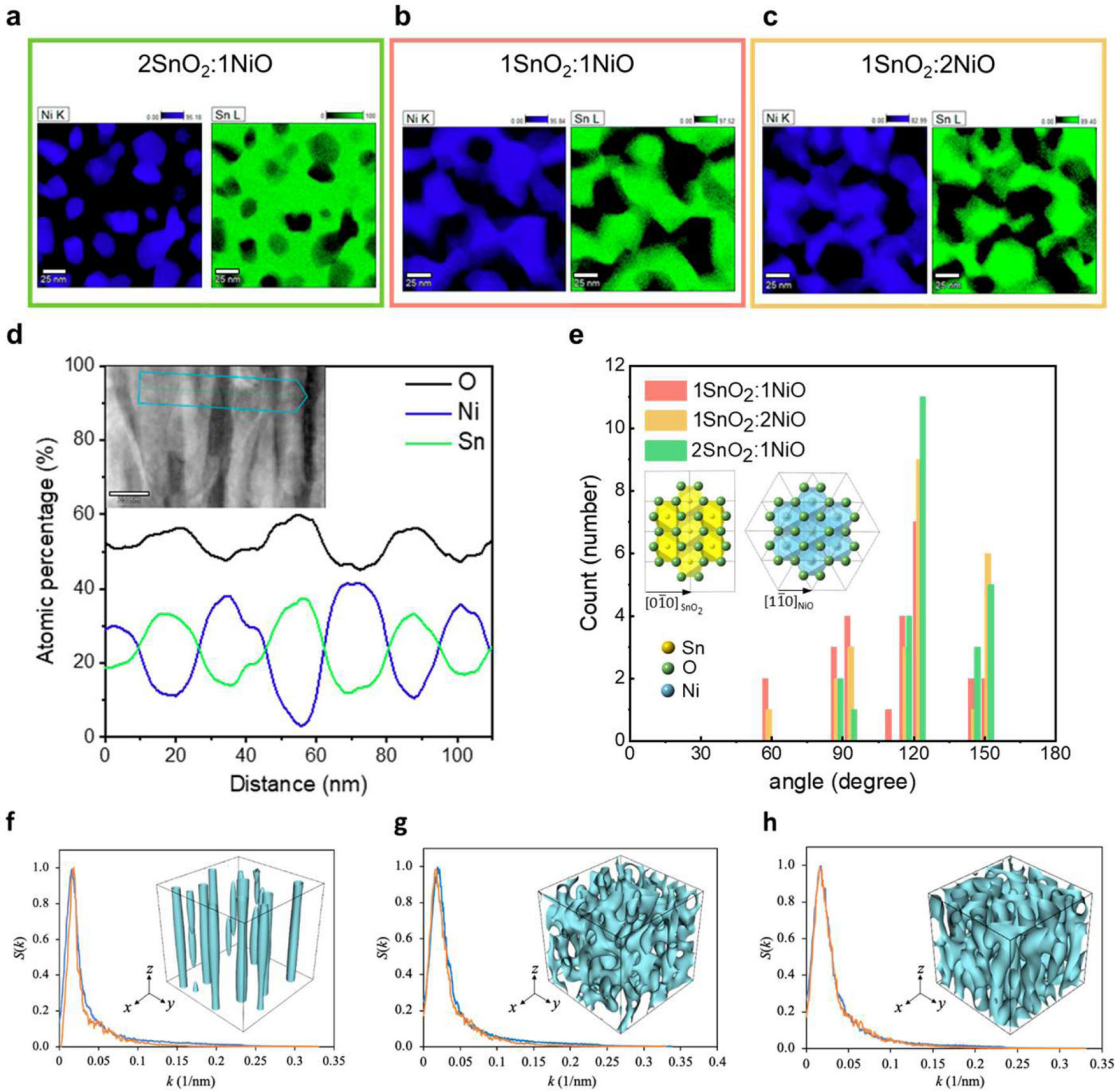
Experimental and estimated nanostructure of the SnO_2_:NiO system. Plane-view TEM-EDS element mapping of nickel (blue) and tin (green) of **a** 2SnO_2_:1NiO, **b** SnO_2_:NiO, **c** 1SnO_2_:2NiO. **d** Compositional distribution of oxygen, nickel, and tin from the cross-sectional TEM image of the SnO_2_-NiO system with a composition ratio of 1:1 (bicontinuous) with the zone axis of *[120]*_*sapphire*_. **e** Distribution of angles at each interface of **a**–**c**. Inset: projection of rutile SnO_2_ and rock salt NiO along the out-of-plane direction. **f**–**h**. Structure factor *S* as a function of wavenumber *k* for the microstructures of 2SnO_2_:1NiO, SnO_2_:NiO, and 1SnO_2_:2NiO, respectively. The blue and orange curves were calculated based on their plane-view TEM images (Supplementary Fig. 8) and the 2D slice of the reconstructed 3D microstructures (shown in the insets, where the cyan color represents the morphology of the NiO phase; size: 256 × 256 × 256 nm).

Reflection high-energy electron diffraction (RHEED) was employed during the deposition of the SnO2:NiO heterostructure to explore the initial growth dynamics and understand the phase separation process in real time. Figure 2a reveals the initial RHEED patterns of the c-plane sapphire substrate along *[120]*_*sapphire*_, and the sharp striped pattern indicates a smooth and ordered surface. Figure 2a-1 reveals the corresponding diffraction pattern and the schematics of sapphire. Figure 2b, c were taken after 75 and 125 pulses, indicating striped patterns, which means that the initial stage of SnO_2_:NiO deposition follows the Frank–van der Merwe growth mode (i.e., layer-by-layer growth). In addition, the diffraction patterns correspond to rock salt NiO (Fig. 2b-1, c-1), and we believe this is the initial unstable structure of the (Ni,Sn)O_x_ solid solution because the exact chemical composition (SnO_2_:NiO) is transferred to the substrate during the deposition process. In addition, Fig. 2d, e reveal the RHEED patterns after 175 and 225 pulses of deposition, indicating the transition from the striped pattern to the spotty pattern. In contrast, the newly formed diffraction patterns in Fig. 2d correspond to rutile SnO_2_ (301) (Fig. 2d-1). This phenomenon indicates that the subsequent deposition of SnO_2_:NiO follows the Stranski–Krastanov (SK) growth mode (i.e., 2D to 3D growth). Moreover, the broad diffraction points in Fig. 2e (marked in orange dotted circles) could be explained by the close diffraction points of SnO_2_ (600) and NiO (22$\bar{4}$) in Fig. 2e-1. This means that above the critical thickness, the rutile structure of SnO_2_ precipitates from the unstable (Ni,Sn)O_x_ phase, which is further confirmed by the as-grown cross-sectional TEM (Supplementary Fig. 6). Therefore, the first decomposition occurs during the fabrication process. In contrast, the second decomposition occurs during the postannealing process (as discussed in Fig. 3).

The rock salt structure tends to deposit first and is followed by precipitation of the rutile structure, which can be attributed to the differences in surface energy and shear modulus. To extend our understanding of this system, a series of density functional theory (DFT) simulations were performed to elucidate the interaction of atomic tin (nickel) with the NiO (SnO_2_) surface. The results of the surface formation energies of Sn_ad_/Ni-terminated NiO(111), Sn_ad_/O-terminated NiO(111), Ni_ad_/Sn-terminated SnO_2_(100), and Ni_ad_/O-terminated SnO_2_(100) models are described in Supplementary Fig. 7, showing that Sn_ad_/O-terminated NiO(111) has the lowest overall energy, even with negative surface energies at chemical potentials corresponding to $\triangle {\mu }_{{Sn}}$ = 0 (Sn-rich) irrespective of the chemical potential condition of $\triangle {\mu }_{{Ni}}$. Note that our calculated surface energies of Sn_ad_/O-terminated NiO(111) are − 0.06 eV/Å^2^ in the Sn-rich and Ni-rich limits, i.e., at $\triangle {\mu }_{{Sn}}$ = 0 and $\triangle {\mu }_{{Ni}}$ = 0, and − 0.13 eV/Å^2^ in the Sn-rich and O-rich limits, i.e., at $\triangle {\mu }_{{Sn}}$ = 0 and $\triangle {\mu }_{{Ni}}$ = −1.13 eV. NiO(111) has a low surface energy, representing preferential growth with the Frank–van der Merwe growth mode. When Sn atoms approach and adsorb on the NiO(111) surface, the surface energy of NiO(111) fluctuates, forming a negative surface energy; the NiO(111) surface becomes rough, and the surface area increases. The driving force due to Sn atoms attaching to the NiO(111) surface causes the nucleation to grow SnO_2_(100). Due to the high surface energy of SnO_2_(100) relative to the surface energy of NiO(111), SnO_2_(100) is grown in three-dimensional (3D) islands in heteroepitaxial growth with the SK growth mode. The surface energy fluctuation will break the crystal symmetry, resulting in surface-directed decomposition.

The determining factors of the morphology in the decomposition process include (a) the degree of compositional fluctuation; (b) the modulus mismatch and the crystal anisotropy between constituent phases; and (c) the lattice mismatch between the film and substrate^22–28^. First, as discussed in Fig. 2, (Ni,Sn)O_x_ is formed in a rock salt structure below the critical thickness, and SnO_2_ with a rutile structure precipitates after reaching the required thickness, displaying the initial phase separation (i.e., first decomposition process) dominated by the lattice mismatch between the substrate and film and the surface energy difference between SnO_2_ and NiO. Second, by controlling the SnO_2_:NiO content to be 2:1, 1:1, and 1:2, the degree of compositional fluctuation can be modulated. The decomposition in SnO_2_:NiO with pillar (Fig. 3a) and bicontinuous (Fig. 3b, c) microstructures could be observed after the postannealing process (i.e., second decomposition process) in the plane-view TEM-EDS with the distribution of nickel and tin. During this procedure, the interface with a compositional gradient between the two phases migrates at the nanoscale, transforming from the as-grown SnO_2_ precipitate (TEM in Supplementary Fig. 6) to pillar or bicontinuous textures (TEM in Supplementary Fig. 2). In addition, this bicontinuous structure represents multiple interfaces beyond the coherent relationship observed in X-ray diffraction, in which the termination of the NiO pillar is surrounded by adjacent SnO_2_, ruling out a pure nucleation and growth mechanism. In addition, the magnified TEM image at the interface (Fig. 3d) with the compositional distribution of nickel and tin along the yellow arrow indicates the compositional gradient rather than a sharp interface. Moreover, these interfaces (Fig. 3a–c) display specific angles. As investigated by the edge detection and Hough transformed method in Supplementary Fig. 7, the angle dependence of interfaces is summarized in Fig. 3e, displaying high intensity at 60°, 90°, 120°, and 150°. This phenomenon indicates that the interface energy still modifies the second decomposition process, which the occupation site in oxides could estimate. The inset of Fig. 3e displays hexagons from the projection of both octahedrons. For rutile SnO_2_ and rock salt NiO, tin and nickel occupy the octahedral sites surrounded by oxygen, indicating that both are coordinated to six oxygen atoms. However, they display, unlike crystal symmetry. Therefore, we believe that this similarity between structures and surface energy fluctuations provides a lower interface energy and dominates the phase separation process, resulting in a bicontinuous design with a specific angle of interfaces.

The thermodynamic driving force for phase separation is typically determined by reducing the local chemical free energy at the expense of the energy penalty from the interface (i.e., the interphase boundary) formation. For solid systems, long-range elastic interactions can also contribute significantly to the driving force. Despite such complexity, the key morphological features of the equilibrium microstructure are primarily dictated by the magnitudes and orientational anisotropy of the specific interface energies *γ*_*i*_ (*i* = *x*,*y*,*z*), where the subscript refers to the axis of the interface normal. This can be understood based on the well-known Wulff construction: a lower interface energy of a crystal leads to a shorter vector length at thermodynamic equilibrium. For example, the formation of vertical NiO pillars in the 2SnO_2_:1NiO sample (Fig. 3a) suggests that *γ*_z_ is significantly larger than both *γ*_*x*_ and *γ*_*y*_, and the pillars are more or less circular, indicating that *γ*_x_≈*γ*_y_. Furthermore, the number of NiO pillars is related to the magnitude of *γ*_*x*_ and *γ*_y_: more pillars tend to form in the case of smaller *γ*_*x*_ and *γ*_y_. For the SnO_2_:NiO and 1SnO_2_:2NiO samples, one can likewise deduce that (1) *γ*_x_≈*γ*_y_ since their microstructure patterns are essentially symmetric along *x* and *y*, as seen from their plane-view TEM images (Supplementary Fig. 9e, h); (2) *γ*_z_ is more significant than *γ*_*x*_ and *γ*_y_ due to the preferred phase alignment along *z*, as shown by the cross-sectional TEM image (see Supplementary Fig. 2). Informed by these TEM images and the proposed phase diagram (Fig. 3d), we performed phase-field simulations to reconstruct the 3D microstructure of the 2SnO_2_:1NiO, SnO_2_:NiO, and 1SnO_2_:2NiO nanocomposites (see Methods). As shown in Fig. 3f–h, the reconstructed microstructure is statistically equivalent to the 2D TEM images at all three NiO concentrations. Such reconstruction allows us to quantify the interface energy anisotropy for the SnO_2_-NiO system and extract key thermodynamic parameters for predicting the microstructure morphologies of SnO_2_-NiO nanocomposites of other NiO concentrations as well as similar oxide nanocomposites. Details are discussed in Supplementary Note 1.

**Fig. 4:**
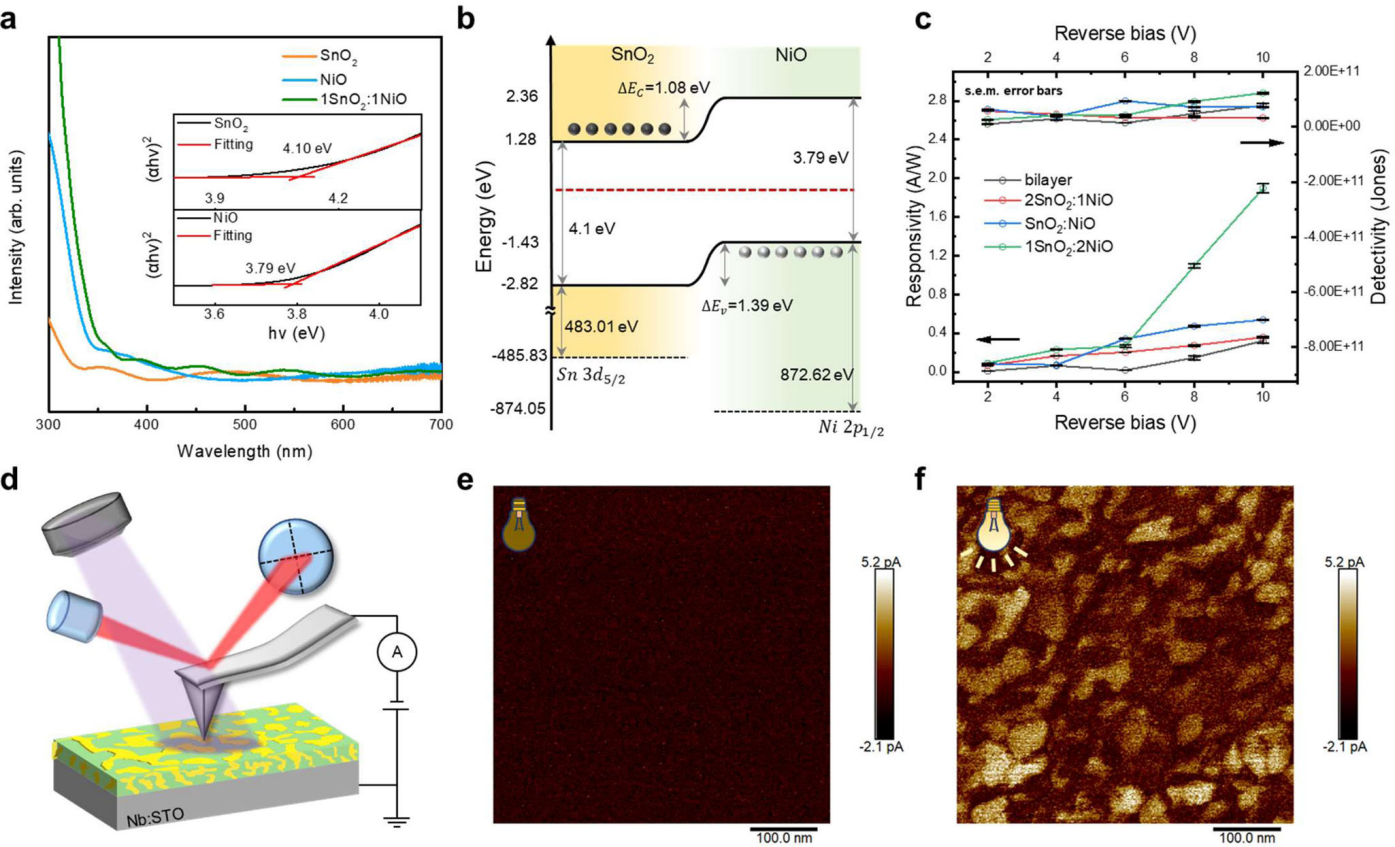
Band structure and photovoltaic properties of SnO_2_:NiO. **a** The absorption spectra of SnO_2_, NiO, and SnO_2_:NiO. Inset: bandgap estimated from the Tauc method. **b** Band diagram estimated by XAS, XPS, and absorption spectrum. **c** Photovoltaic response of annealed SnO_2_:NiO with a composition ratio of 2:1 (pillar), 1:1 (bicontinuous) 1:2 (bicontinuous), and bilayer SnO_2_/NiO. **d** Illustration of photoconductive AFM on a SnO_2_:NiO/Nb:STO thin film with a composition ratio of 1:1 (bicontinuous). The external laser source with a 405 nm wavelength is coconstructed with a commercial AFM system. **e** Dark current, and **f** photocurrent of the SnO_2_:NiO/Nb:STO thin film under a 10 V sample bias.

After a detailed exploration of the structure, attention is given to charge interactions at the interface. The photoelectric conversion process includes light absorption, photocarrier generation, photocarrier transportation, and photocarrier extraction. A heterostructure’s photovoltaic and photoconductive responses are typically enhanced by forming a p-n junction with proper band alignment. The effective interaction depends on the carrier concentration of p-type and n-type materials, creating a depletion region. The advantage of such a bicontinuous structure is the formation of depletion regions in the whole sample, and the control of thickness can achieve optimized light absorption. The composition and thickness can be two independent factors for optimization; unlike a p-n junction in the bilayer structure, they are coupled. Therefore, with the p-n heterojunction and a high interface-to-volume ratio, the bicontinuous structure of SnO_2_:NiO holds the advantage and potential to display a prominent photocurrent (i.e., photovoltaic effect). First, the bandgaps of SnO_2_ and NiO are estimated to be 4.10 eV and 3.79 eV based on the spectra of optical absorption and the extrapolation from the Tauc method in the inset of Fig. 4a, indicating that the SnO_2_:NiO heterostructure could absorb near UV illumination. Second, the band diagram of SnO_2_:NiO could be characterized by a combination of the valance band maximum and the binding energy extracted from X-ray photoelectron spectroscopy (Supplementary Fig. 11) and bandgap analysis, as summarized in Fig. 4b. Due to the band bending in the type-II heterostructure, the photogenerated electrons would accumulate at SnO_2_. At the same time, the holes would assemble at NiO. In addition, the depletion region was calculated to be ~ 29.8 nm (in Supplementary Note 2), which is comparable to the domain size in a bicontinuous structure (Fig. 3a–c). Therefore, an efficient carrier transport path could be expected regardless of the film thickness with a high interface-to-volume ratio. In Fig. 4c, the photovoltaic effect of the SnO_2_/NiO bilayer, as well as the pillar and bicontinuous textures of self-assembled SnO_2_:NiO, could be detected under the illumination of UV light with a wavelength of 265 nm, whose photon energy is more significant than the bandgaps of SnO_2_ and NiO. The nonlinear photocurrent–voltage relationship (in Supplementary Fig. 12) reveals Schottky contact with a breakdown voltage of 26 V, representing typical photodiode behavior. Therefore, the depletion region and photocurrent could be modulated by transverse bias, and the responsivity (*R*) and detectivity (*D*) could be calculated by the following equations:

1 $${{{{{\rm{R}}}}}}=\frac{{J}_{{{{{{\rm{light}}}}}}}-{J}_{dark}}{{{{{{{\rm{P}}}}}}}_{in}}$$

2 $${{{{{\rm{D}}}}}}\ast \cong \frac{{{{{{\rm{R}}}}}}}{\sqrt{2q{J}_{dark}}}$$

*J*_*light*_, *J*_*dark*_, P, and q represent the photocurrent density (A × cm^−2^), dark current (A × cm^−2^), photo intensity (W/cm^2^), and elementary charge (i.e., 1.602 × 10^−19^ C), respectively. As shown in Fig. 4c, the bilayer structure displays the lowest responsivity at each bias. In contrast, 1SnO_2_:2NiO with a bicontinuous structure reaches a maximum responsivity of 2 A/W at a 10 V bias, and the detectivity reaches 10^11^ Jones, indicating that the photoactive heterojunction holds immense potential for photodetectors. On the other hand, we can obtain an adequate thickness of light absorption to enhance performance, which is determined to be 250 nm (discussed in Supplementary Fig. 13). In addition, the wavelength (i.e., domain size) of the bicontinuous SnO_2_:NiO system is comparable to the depletion length calculated from the Hall effect measurement in Supplementary Note 2, which implies that the depletion region extends through the whole thin film, explaining why the bicontinuous structure displays higher responsivity than the bilayer structure.

The photoconductive response reveals that the interface-to-volume ratio of SnO_2_:NiO dominates the current transport mechanism. Therefore, photocurrent detection through the AFM technique could build a close relationship between the photocurrent and the nanocomposite microstructure. Figure 4d demonstrates the schematic diagram of the measurement. An external laser illumination source with a wavelength of 405 nm was installed with a commercial AFM system, and the current was collected under a 10 V sample bias. Compared with the dark current in Fig. 4e with the same scale, the photocurrent as imaged in Fig. 4f displays a significant photocurrent with the bicontinuous feature, which could be compared with the plane-view TEM with the self-organized feature in Supplementary Fig. 14. In addition, the higher photocurrent area is expected to be a SnO_2_-rich region due to the higher mobility and higher carrier concentration^29,30^. Therefore, based on the photovoltaic and localized photocurrent distribution analysis, the self-assembled structure with more extensive regions of p-n interfaces displays higher responsivity and detectivity, delivering promising evidence of the strong charge interaction of the bicontinuous structure.

## Discussion

This study demonstrates a self-assembled oxide with a bicontinuous structure in the SnO_2_:NiO system. The in situ growth mechanism was monitored by RHEED, indicating that a rock salt structure with a minor misfit formed initially and then was followed by the precipitation of a rutile structure with a more significant misfit. In addition, decomposition occurred during the annealing process. XRD and TEM have identified the epitaxial relationship, while the elemental distribution of Sn and Ni reveals a bicontinuous structure with a specific angle-dependent interface. Although rutile SnO_2_ and rock salt NiO display different crystal symmetry and oxidation states, we suggest the bicontinuous form because their similar projected octahedral frameworks could be considered a continuous expansion of coherent hexagonal atomic arrangements in sapphire. Benefiting from a higher interface-to-volume ratio, the photovoltaic activity of the bicontinuous structure is more potent than that of the bilayer structure, revealing the advantage of a tortuous three-dimensional nanocomposite with strong charge interactions. This work displays a double-percolated system with a distinct structure in oxide systems. This expands the practical application of self-assembled oxide systems through well-designed electronic interfaces in VANs.

## Methods

### Sample preparation and annealing process

The SnO_2_:NiO/sapphire, SnO_2_/sapphire, and NiO/sapphire thin film was fabricated via pulsed laser deposition^31^ with commercial SnO_2_:NiO (SnO_2_: NiO = 1:1, 1:2, 2:1), SnO_2_, NiO, and ITO (90% In_2_O_3_ and 10% SnO_2_) targets (ITO is for a top electrode in photo-voltaic analysis). Commercial c-sapphire was used as the substrate. Before the deposition process, the vacuum chamber was evacuated to a base pressure of 1 × 10^−5^ Torr. SnO_2_ was deposited at 3 mTorr, and the substrate was heated to 750 °C, while NiO was grown under the same atmosphere, and the substrate was cooled down to 550 °C. The growth of SnO_2_:NiO was carried out at 8 mTorr with a substrate temperature of 750 °C. Lastly, ITO top electrode (30 nm) was deposited by RF sputtering at 0.3 mTorr with an Ar flow of 20 sccm under room temperature. The RF power was controlled at 175 W. The annealing process is operated at 900 °C for 6 hrs, followed by the cooling rate of 0.4 °C/s. The thickness of the thin film is ~250 nm.

### X-ray diffraction and absorption spectrum

XRD investigation was carried out by Bruker D2 Discover XRD System with Cu K_α_ x-ray (*λ* = 1.5406 Å) to acquire the 2θ-θ scan along a normal direction. *φ* scans and rocking curve measurements were acquired using a D8 advance XRD (Bruker). The optical absorbance of the heterostructure was measured by a commercial PerkinElmer Lambda-900 spectrometer.

### Electron microscopy characterization and analysis

The specimen was prepared by focused ion beam (FEI Helios NanoLab 660) for TEM analyses. The ion-beam source’s resolution can reach 4 nm at 30KV accelerating voltage. The information of HRTEM and STEM-EDS were acquired at room temperature on (JEOL JEM-2800F) with 200KV accelerating voltage. The TEM and STEM resolutions were 0.1 nm and 0.2 nm, respectively.

### First-principles DFT calculations

We studied the surface formation energy of atomic tin with Ni- and O-terminated NiO(111) and atomic nickel with Sn- and O-terminated SnO_2_(100) by first-principles DFT calculations with general gradient approximation (GGA) functional PW91^32,33^, implemented in the Vienna ab initio simulation package (VASP) code^34–36^. The electronic configurations for valence electrons are O 2*s*^2^2*p*^4^, and Ni 4*s*^2^3*d*^8^, and Sn 5*s*^2^5*p*^2^. The converged slab geometries of one tin (nickel) atom on the Ni- and O-terminated NiO(111) (Sn- and O-terminated SnO_2_(100)) separated by a vacuum region equivalent to a thickness of 40 Å, i.e., Sn_ad_/Ni-terminated NiO(111), Sn_ad_/O-terminated NiO(111), Ni_ad_/Sn-terminated SnO_2_(100), and Ni_ad_/O-terminated SnO_2_(100) models, were constructed using bulk crystalline configurations. The surface formation energy σ is defined as the excess energy on the surface of a slab compared with the bulk, namely, σ = (*E*_*slab*_− $\mathop{\sum}\limits_{i}{n}_{i}{\mu }_{i}$) / 2 *A*, where *E*_*slab*_ is the total energy of the slabs with repeated geometry. *n*_*i*_ and *μ*_*i*_ are the numbers of atoms and chemical potentials of the constituents of the slab, respectively. *A* is the surface area. The chemical potential of Ni is restricted within the thermodynamically allowed ranges as follows: ${\mu }_{{Ni}}^{{bulk}}$ + ∆*H*^ *f*^ [*NiO*] ≤ *μ*_*Ni*_ ≤  ${\mu }_{{Ni}}^{{bulk}}$, i.e. ∆*H*^ *f*^ [*NiO*] ≤ *μ*_*Ni*_ −${\mu }_{{Ni}}^{{bulk}}$ ≤ 0 or simply ∆*H*^*f*^ [*NiO*] ≤ $\triangle {\mu }_{{Ni}}$ ≤ 0, the chemical potential of Sn is restricted within the thermodynamically allowed ranges as follows: ${\mu }_{{Sn}}^{{bulk}}$ + ∆*H*^ *f*^ [*SnO*_2_] ≤ *μ*_*Sn*_ ≤ ${\mu }_{{Sn}}^{{bulk}}$, i.e. ∆*H*^ *f*^ [*SnO*_2_] ≤ ${\mu }_{{Sn}}$− ${\mu }_{{Sn}}^{{bulk}}$ ≤ 0 or simply ∆*H*^ *f*^ [*SnO*_2_] ≤ $\triangle {\mu }_{{Sn}}$ ≤ 0. Our formation enthalpy of bulk NiO and SnO_2_, ∆*H*^ *f*^ [*NiO*] and ∆*H*^ *f*^ [*SnO*_2_], is determined from the total energies per atom of bulk NiO (space group: 225 *Fm-3m*) and bulk SnO_2_ (space group: 136 *P4*_*2*_ */mnm*), respectively, yielding ∆*H*^ *f*^ [*NiO*] = −1.13 eV and ∆*H* ^*f*^ [*SnO*_2_] = −5.30 eV, in excellent agreement with other calculated value of ∆*H* ^*f*^ [*SnO*_2_] = −5.90 eV^37^.

### Phase-field simulations

The equilibrium phase morphologies of the self-assembled SnO_2_-NiO nanocomposite films were simulated using a 3D phase-field model of phase separation^38^ implemented in the μ-Pro® software package. Both kinetic pathways of phase separation: nucleation and growth, and near-spinodal decomposition are considered depending on the concentration of NiO. Each microstructure is described using the 3D spatial distribution of the concentration of NiO (*c*_NiO_) in the two-phase mixture. The equilibrium distribution of *c*_NiO_ was obtained by numerically solving the Cahn-Hilliard equation without a priori assumption on the microstructure patterns. 2D Fast Fourier Transformation is performed to extract the feature lengths of the two-phase microstructure from each 2D slice of the 3D microstructure pattern for comparison to those extracted the same way from the TEM images. Details of the phase-field simulations and feature-length extraction are provided in Supplementary Note 1.

### Photo-conductive atomic force microscopy

Photo-conductive atomic force microscopy was carried out on a commercial SPM system (Bruker MultiMode 8 SPM system) with the combination of the light source of a 405 nm laser pointer. Diamond tips (Adama AD-40-AS, resonance frequency = 200 kHz) were used for photo-current detection.

### Photo-voltaic measurement

To conduct the photovoltaic measurement, the ITO top circular electrode (radius= 100 μm) was deposited via a mask by RF sputtering, and the bottom electrode was further connected to form a circuit. When applying bias, to remain within the reverse bias region, 80 nm of NiO (p-type) thin film was deposited above the bicontinuous and pillar structure of the SnO_2_-NiO system. The dark and photocurrent measurements were carried out by a semiconductor analyzer (Keithley 4200 and Keysight B1500a) with the combination of a light source under a wavelength of 265 nm and an energy density of 0.375 mW cm^−2^, and the current density was obtained about the top electrode area (~3.14 × 10^−4^ cm^2^).

## Supplementary information

Supplementary Information

## Data Availability

All data supporting the results of this study are available in the manuscript or the supplementary information. Additional data are available from the corresponding author upon request.
