# Publisher Correction: Bicontinuous oxide heteroepitaxy with enhanced photoconductivity

**DOI:** 10.1038/s41467-023-35885-7

**Published:** 2023-01-12

**Authors:** Pao-Wen Shao, Yi-Xian Wu, Wei-Han Chen, Mojue Zhang, Minyi Dai, Yen-Chien Kuo, Shang-Hsien Hsieh, Yi-Cheng Tang, Po-Liang Liu, Pu Yu, Yuang Chen, Rong Huang, Chia-Hao Chen, Ju-Hung Hsu, Yi-Chun Chen, Jia-Mian Hu, Ying-Hao Chu

**Affiliations:** 1 Department of Materials Science and Engineering, National Yang Ming Chiao Tung University, Hsinchu, 30010 Taiwan; 2 Department of Materials Science and Engineering, University of Wisconsin-Madison, Madison, WI 53706 USA; 3 National Synchrotron Radiation Research Center, Hsinchu, 30076 Taiwan; 4 Graduate Institute of Precision Engineering, National Chung Hsing University, Taichung, 402 Taiwan; 5 State Key Laboratory of Low Dimensional Quantum Physics and Department of Physics, Tsinghua University, 100084 Beijing, People’s Republic of China; 6 Key Laboratory of Polar Materials and Devices, Ministry of Education, East China Normal University, 200241 Shanghai, China; 7 Integrated Service Technology, Hsinchu, Taiwan; 8 Department of Physics, National Cheng Kung University, Tainan, 70101 Taiwan; 9 Department of Materials Science and Engineering, National Tsing Hua University, Hsinchu, 30013 Taiwan

**Keywords:** Nanoscale materials, Materials for devices

Correction to: *Nature Communications* 10.1038/s41467-022-35385-0, published online 03 January 2023

The original version of this Article incorrectly made the peer review reports for this paper available. In the correct HTML version, the link to the peer review file has been removed.

