# Supplementary material for: Bicontinuous oxide heteroepitaxy with enhanced photoconductivity
Source: Nat Commun. 2023 Jan 3;14:21. doi: 10.1038/s41467-022-35385-0 (PMC9810741; doi:10.1038/s41467-022-35385-0)
Supplement: Supplementary file 1 — Supplementary Information [file 41467_2022_35385_MOESM1_ESM.pdf]

SUPPLEMENTARY INFORMATION

Bicontinuous Bulk Oxide Heteroepitaxy with Enhanced Photoconductivity

Pao-Wen Shao[1], Yi-Xian Wu[1], Wei-Han Chen[1], Mojue Zhang[2], Minyi Dai[2], Yen-Chien Kuo[3], Shang-Hsien Hsieh[3], Yi-Cheng Tang[4], Po-Liang Liu[4], Pu Yu[5], Yuang Chen[6], Rong Huang[6], Chia-Hao Chen[3], Ju-Hung Hsu[7], Yi-Chun Chen[8], Jia-Mian Hu[2*] and Ying-Hao Chu[1,9*]

*Correspondence to: jhu238@wisc.edu and yhchu@mx.nthu.edu.tw

This file includes:

Supplementary Notes 1-2

Supplementary Figures 1-14

Supplementary Tables 1-4

Supplementary Note and Figures

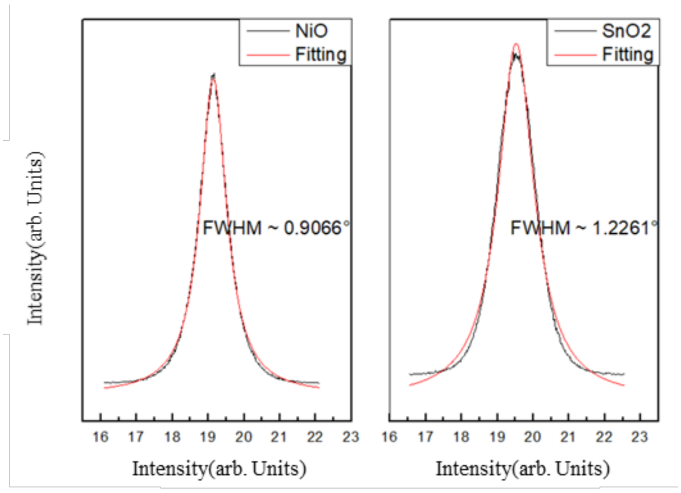

Supplementary Fig. 1 NiO(111) and SnO_2(200) Rocking curves.

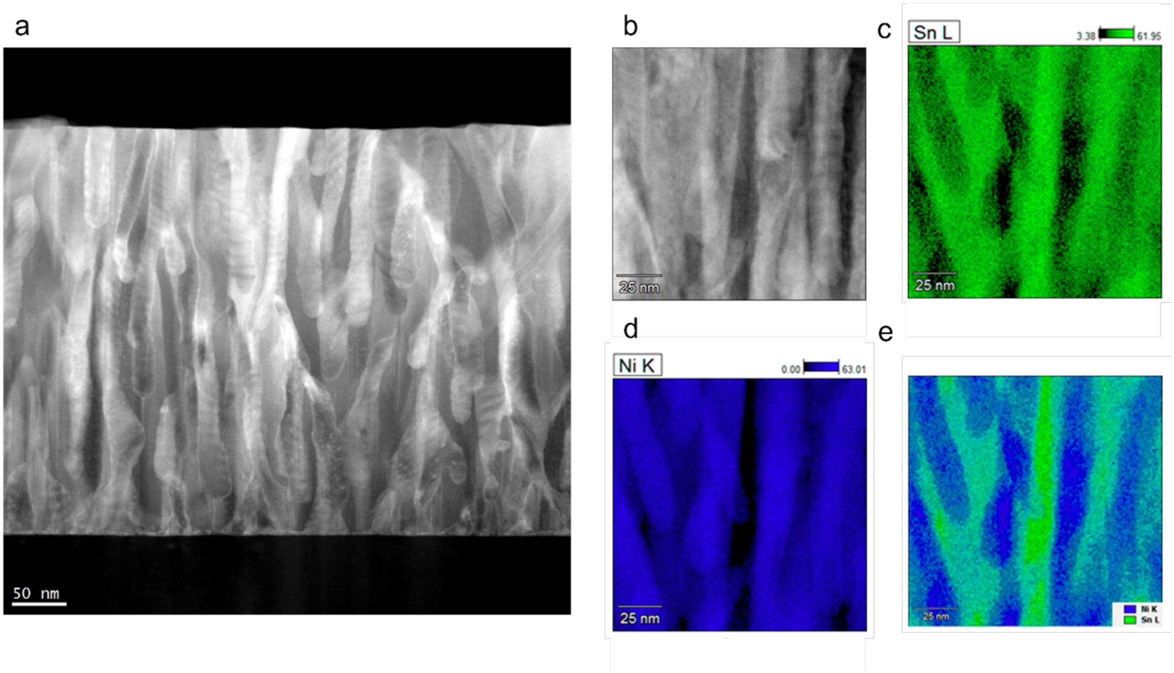

Supplementary Fig. 2 STEM and EDS image of SnO_2:NiO with the composition of 50 % Ni after post-annealing.

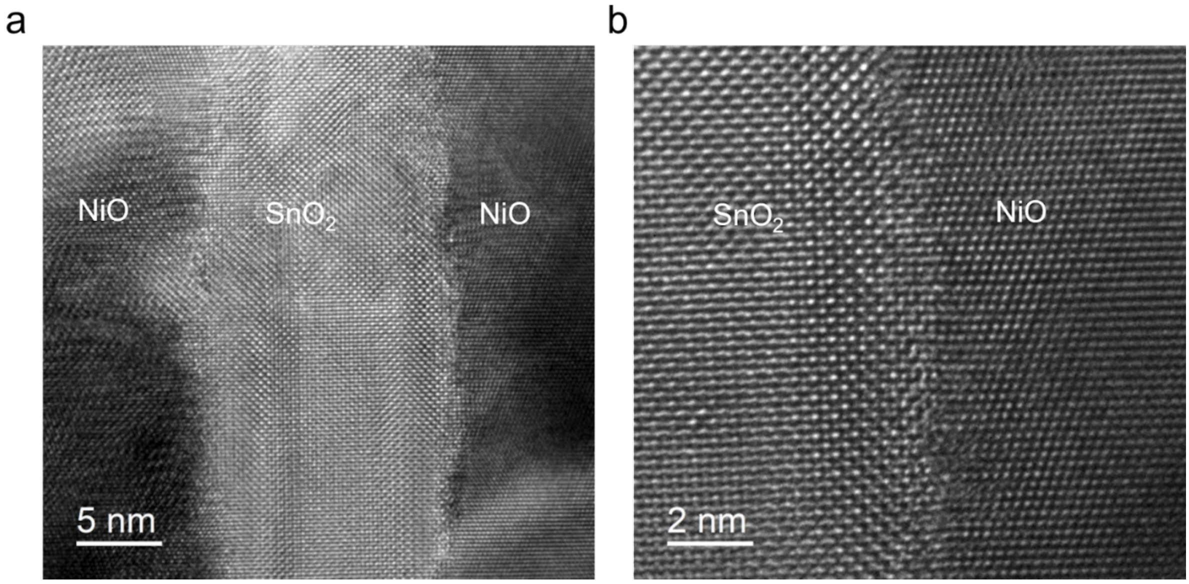

Supplementary Fig. 3 a, b HRTEM observation of the SnO_2/NiO interface in the bi-continuous sample. The zone axis is $[120]_{sapphire}$.

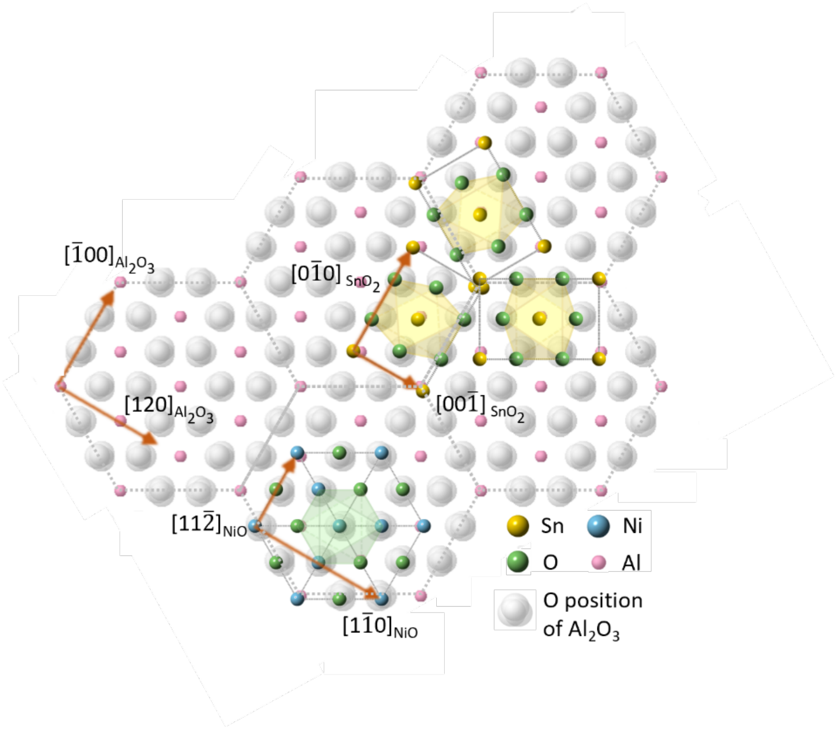

Supplementary Fig. 4 Lattice projection of (SnO_2:NiO) along epitaxial axis (*$[400]_{SnO2}||[111]_{NiO}||[001]_{sapphire}$*). The orange arrows represent the in-plane orientation relationship. The projected positions of oxygen octahedrons in SnO_2 and NiO overlap with

that of sapphire.

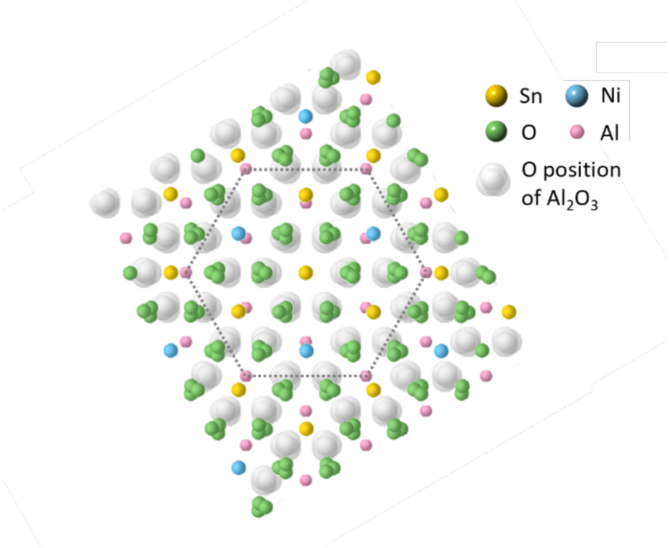

Supplementary Fig. 5 Imagined lattice projection of $NiSnO_3$ along *[001]*$_{NiSnO3}$ and *[001]*$_{sapphire}$. The Sn and Al atoms at the center of the hexagon are overlapped. The large misfit (2.6 %) between $NiSnO_3$ and sapphire suggests that the $NiSnO_3$ formation is not the energetically favorable.

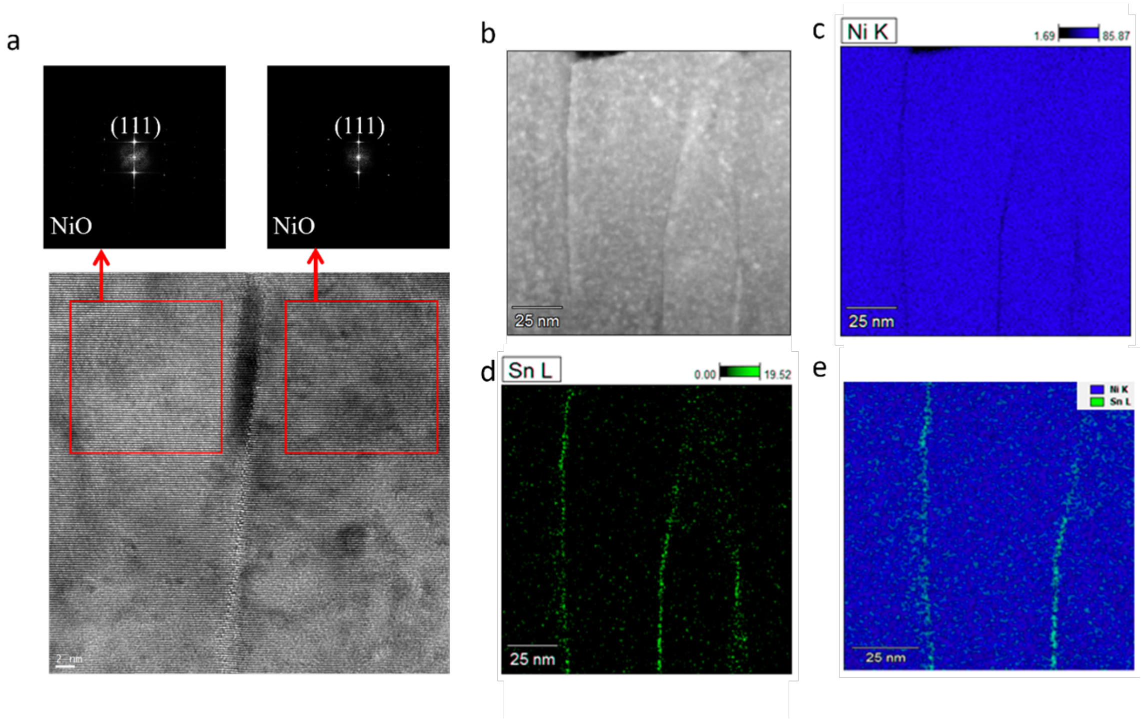

Supplementary Fig. 6 a TEM and corresponding FFT of as-grown SnO_2:NiO. **b-e** STEM EDS analysis with the elemental distribution of Ni and Sn. The first decomposition process occurs during fabrication, showing that SnO_2 precipitates at the domain wall of NiO.

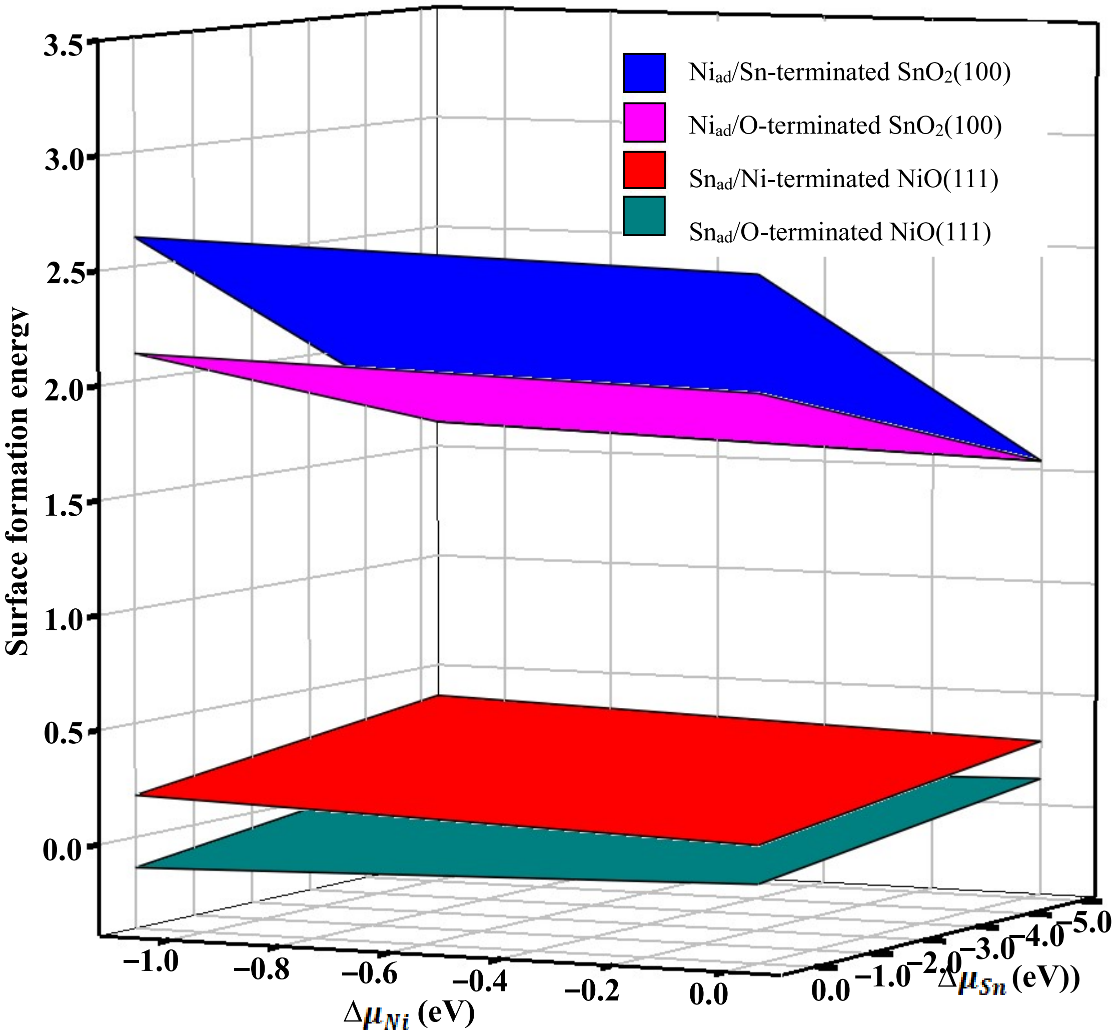

Supplementary Fig. 7 3D plot of surface formation energies of Sn$_{ad}$/Ni-terminated NiO(111), Sn$_{ad}$/O-terminated NiO(111), Ni$_{ad}$/Sn-terminated SnO_2(100), and Ni$_{ad}$/O-terminated SnO_2(100) models as bilinear functions of the allowed Ni and Sn chemical potentials. The Sn$_{ad}$/O-terminated NiO(111) model has the lowest surface energy among all four models.

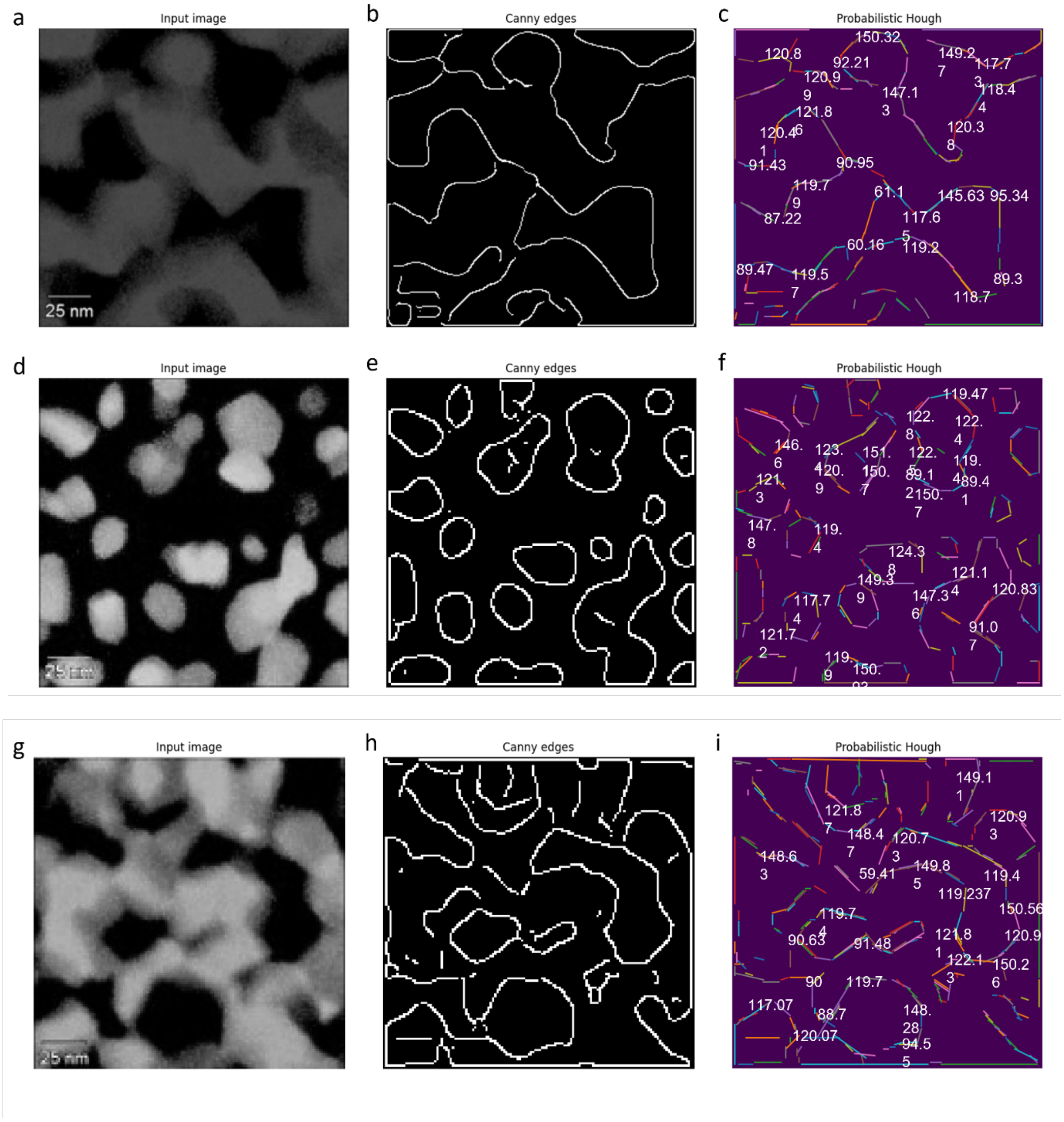

Supplementary Fig. 8 Edge-detecting and geometry-determine technique by Python. The grayscale of the EDS image of **a** SnO_2:NiO, **d** 2SnO_2:1NiO, **g** 1SnO_2:2NiO. Canny-edge detection result of **b** SnO_2:NiO, **e** 2SnO_2:1NiO, **h** 1SnO_2:2NiO. Hough transform result of **b** SnO_2:NiO, **e** 2SnO_2:1NiO, **h** 1SnO_2:2NiO.

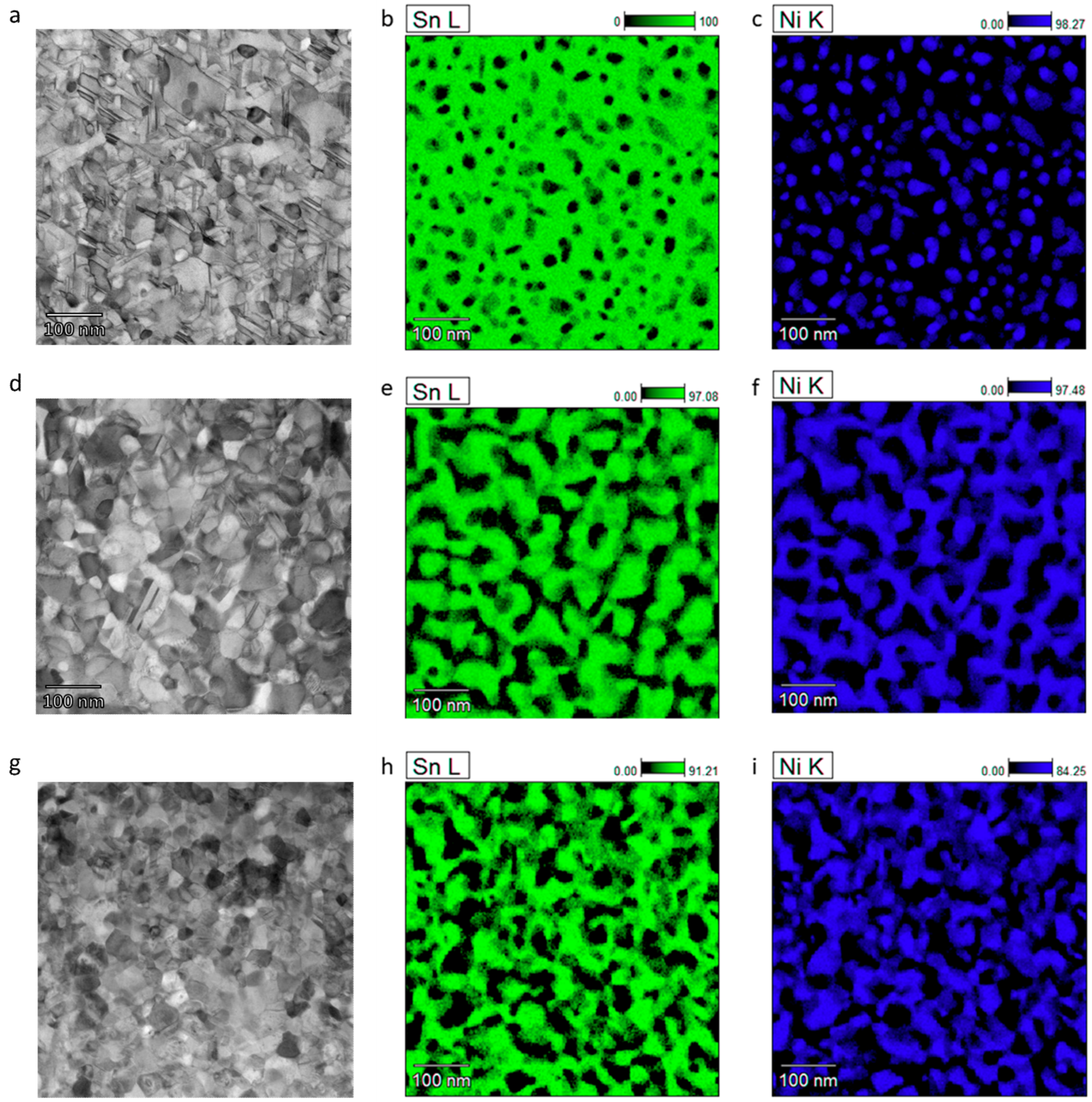

Supplementary Fig. 9 Plane-view TEM (**a**, **d**, **g**) and TEM-EDS element mapping of tin (green) and nickel (blue) of **b, c** $2SnO_2$:1NiO, **e, f** SnO_2:NiO, **h, i** $1SnO_2$:2NiO.

Supplementary Note 1: Details of the phase-field model for phase separation

Phase-field modeling has been routinely used to simulate microstructure evolution during phase separation via spinodal decomposition or nucleation and growth[1, 2, 3]. Here, we perform phase-field modeling to reconstruct the 3D equilibrium microstructure of the 1-$x$$SnO_2$-$x$NiO ($x$=0.33, 0.5, 0.67) nanocomposite thin films based on their plane-view (**Supplementary Fig.**

8) and cross-sectional (**Supplementary Fig. 2**) TEM images.

In the present phase-field model, the equilibrium microstructure is described by the spatial distribution of the local concentration of the NiO, c_{NiO} (denoted as "c" hereafter), in the SnO_2-NiO two-phase mixture. The temporal evolution of the c is governed by the Cahn-Hilliard equation,

$$\frac{\partial c}{\partial t} = \nabla \cdot M_{ij} \nabla_j \frac{\delta F}{\delta c}; i, j = (x, y, z), \quad (1)$$

where F is the total Helmholtz free energy of the two-phase system[4]; M_{ij} is the mobility which should be a function of the mobilities of all the diffusing species as well as their local concentration [3]. For simplicity, we assume $M_{ij} = M(1 - ac^2)$ following refs. [1, 5], where a=0 and a=1 correspond to bulk-diffusion-controlled and interface-diffusion-controlled dynamics, respectively. In the simulations, we set M=1 and a=0.9 to model interface-diffusion-dominated dynamics considering the moderate bulk interdiffusion across the SnO_2-NiO interface. Yet, it is noteworthy that the equilibrium morphology is mainly determined by the competition between the local chemical free energy and the energy of the SnO_2-NiO interface, both of which contribute to the total Helmholtz free energy F. In a diffuse-interface description, F is written as,

$$F = \int_V \left[f(c) + \frac{1}{2} \kappa_{ij} (\nabla_i c)(\nabla_j c) \right] dV. \quad (2)$$

Here $f(c)$ is the local chemical free energy density (J/m^3), which typically takes the form of a double-well potential [2, 3],

$$f(c) = \frac{16w}{(c^\alpha - c^\beta)^4} (c - c^\alpha)^2 \left(c - c^\beta\right)^2, \quad (3)$$

where w is the energy density barrier (J/m^3), c^α and c^β are the limiting concentrations of the NiO and SnO_2 phases, respectively, which defines the miscibility gap ($c^\alpha < c < c^\beta$). The choice of c^α and c^β also determines the boundary concentrations of the spinodal region, at which $\partial^2 f(c)/\partial c^2 = 0$. Furthermore, the selection of the c^α and c^β also affects the thermodynamic driving force for the nucleation of a new phase from a parent phase. For example, the thermodynamic driving force for the nucleation of NiO is evaluated as $\Delta\mu = \frac{\partial f(c)}{\partial c}(c^\beta - c) + f(c)$. A larger thermodynamic driving force leads to a larger nucleation rate and probability, thus facilitating nucleation. In this work, we set c^α=0.25 and c^β=0.9,

yielding a spinodal region of $0.387<c<0.763$. As a result, the microstructure morphology of the $0.67SnO_2$-$0.33NiO$ nanocomposite film was modeled by first forming NiO nuclei in the matrix based on the classical theory of nucleation and then simulating their growth by numerically solving Eq. (1). The microstructures of the $0.5SnO_2$-$0.5NiO$ and $0.33SnO_2$-$0.67NiO$ form via spinodal decomposition, which was modeled by directly solving Eq. (1)

The second term in the integrand of Eq. (2) is the gradient energy density describing the short-range interaction between the diffusing species, where κ_{ij} is the gradient energy coefficient (J/m). The gradient energy density is part of the total interface energy density. Since $f(c)$ is isotropic, the shape anisotropy of the equilibrium microstructure is determined by anisotropy of the specific SnO_2-NiO interface energy (J/m^2), i.e., the γ_x, γ_y, γ_z, as discussed in the main text.

The specific interface energy is proportional to the gradient energy coefficient. In the limit of a 1D system, one can have the analytical solution of $\gamma = \frac{2\kappa}{3\lambda}$ [3], where λ is the interface width. Thus, the anisotropy in the specific interface energy can be considered by parametrizing the diagonal components of the gradient energy coefficient matrix $\kappa_{xx}, \kappa_{yy}, \kappa_{zz}$. If assuming λ~6nm (a rough estimate from Fig. 3d) and considering a specific interface energy γ~0.3 J/m^2 (a typical value for semi-coherent interfaces), one has $\kappa=\kappa_0\sim2.7\times10^{-9}$ J/m (estimated using the formula mentioned above). Since the actual length scale of the phases depends on the both the gradient energy and the local chemical free energy (related to w), we performed high-throughput 3D phase-field simulations[3] to identify the values of κ and w that can lead to microstructures patterns that are both visually and statistically similar to the TEM images at equilibrium. To this end, we numerically solve the Cahn-Hilliard equation (Eq. (1)) in a discrete 3D periodic system using a semi-implicit Fourier spectral algorithm[6] with dimensions of $128\Delta x\times128\Delta y\times128\Delta z$. The grid sizes $\Delta x=\Delta y=\Delta z=l_0=2$ nm, and note that $l_0 = \sqrt{\kappa/w}$ for nondimensionalization. For spinodal decomposition, the initial microstructure is a uniform concentration (c=0.5 or 0.67) superimposed with a sinusoidal concentration wave with a peak amplitude of ~0.02. In the case of nucleation and growth, the initial microstructure was generated based on classical nucleation theory as mentioned above (details of implementation are available in ref. [3]).

To evaluate the structural statistics of the microstructure images, we performed a 2D fast

Fourier transform (FFT) to extract the feature length l^* for every 2D slice of the simulated 3D microstructures and the 2D TEM images. As an example, **Supplementary Fig. 9** below shows the diffraction pattern of the TEM image for the $0.5SnO_2$-0.5NiO sample (**Supplementary Fig. 9f**). From the 2D diffraction pattern, we can calculate the structure factor via the following equation[7],

$$S(k) = \int_{-\pi}^{\pi} \left| \frac{1}{\sqrt{\pi}} \int u(x,y) e^{i\vec{k}\vec{r}} dxdy \right| k d\theta, \tag{4}$$

where $u = u(x,y)$ is the concentration or the RGB density of an image pixel; $k=|\vec{k}|$ and θ are the polar coordinate representation of the wave-vector space k. The numerical implementation of Eq. (4) is to sum the FFT amplitude over circles of radii equal to the reciprocal of the wavelength. Plots of $S(k)$ are shown in Figs. 3f-h in the main text. The feature length l^* is then calculated as $l^*=1/k_{peak}$, where k_{peak} is the wavenumber at which $S(k)$ peaks. This approach has been commonly used to determine the feature length of Labyrinthine patterns[7, 8]. **Supplementary Table 1** summarizes the k_{peak} and feature lengths in Figs. 3f-h and the key thermodynamic parameters utilized in phase-field modeling.

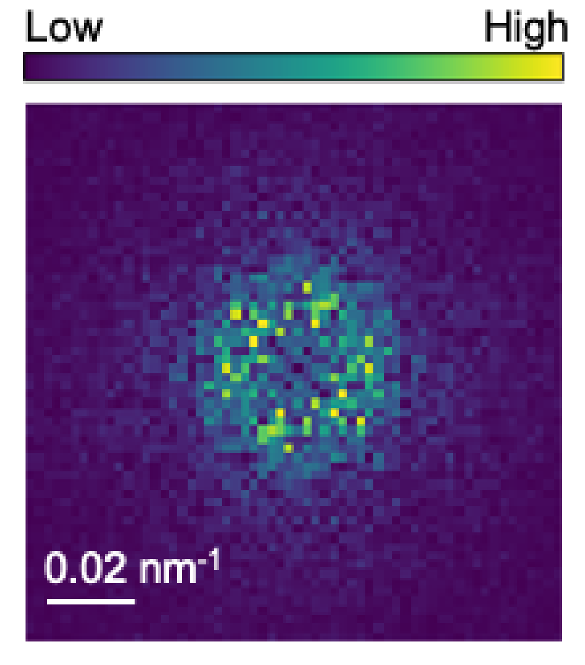

Supplementary Fig. 10 2D FFT of the plane-view TEM image of the $0.5SnO_2$-0.5NiO sample.

Supplementary Table 1 Data associated with Figs. 3g-i and key thermodynamic parameters.

	0.67SnO_2-0.33NiO	0.5SnO_2-0.5NiO	0.33SnO_2-0.67NiO
k_{peak} (nm^{-1})	0.0210 (simulation)<br>0.0216 (experiment)	0.0195 (simulation)<br>0.0197 (experiment)	0.0197 (simulation)<br>0.0198 (experiment)
l^* (nm)	47.52 (simulation)<br>46.25 (experiment)	51.2 (simulation)<br>50.6 (experiment)	50.8 (simulation)<br>50.4 (experiment)
κ_{xx}/κ_0	0.2	0.25	0.25
κ_{yy}/κ_0	0.2	0.25	0.25
κ_{zz}/κ_0	10	0.4	0.4
w (J/m^3)	0.05	0.0625	0.0625

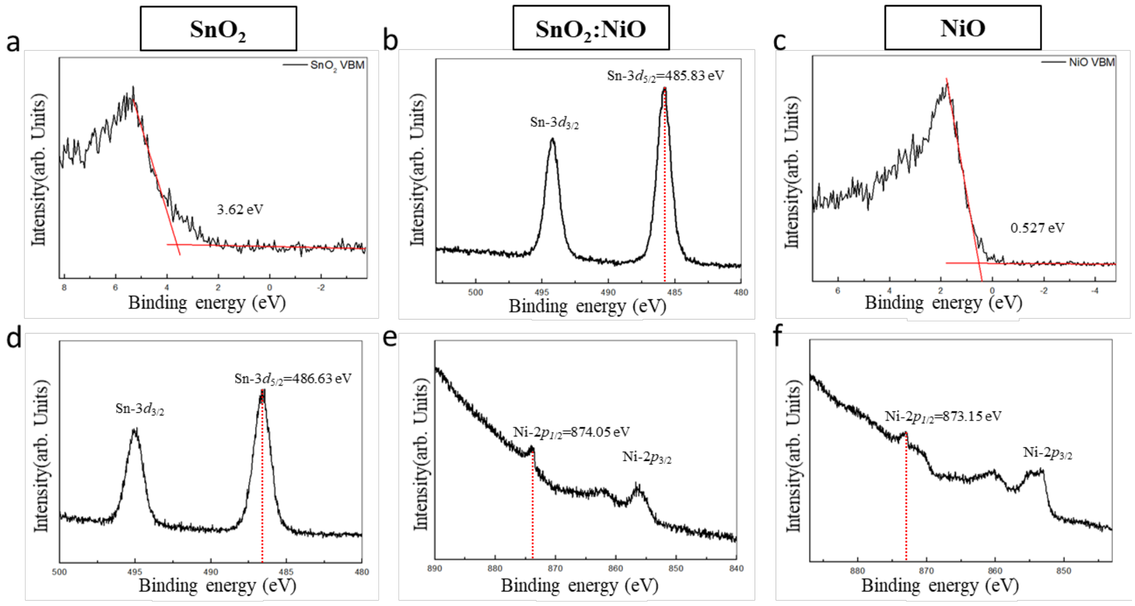

Supplementary Fig. 11 XPS valence band maximum of **a** SnO_2 and **c** NiO. Sn 3*d*-core level spectra of **b** SnO_2:NiO and **d** SnO_2. Ni 2*p*-core level spectra of **e** SnO_2:NiO and **f** NiO.

Supplementary Table 2 Summary of XPS analysis.

	Sn $3d_{5/2}$	Ni $2p_{1/2}$	VBM (valence band maximum)	Sn $3d_{5/2}$-VBM or Ni $2p_{1/2}$-VBM
SnO_2	486.63 eV		3.62 eV	483.01 eV
NiO		873.15 eV	0.53 eV	872.62 eV
SnO_2 : NiO	485.83 eV	874.05 eV		

Supplementary Note 2: Length of the depletion region

According to the band diagram in **Figure 4b**, the built-in potential is 1.08 eV. To investigate the length of the depletion region, Hall effect measurement is applied to NiO and SnO_2. Major carrier concentration is $3.20 \times 10^{18}\ cm^{-3}$ for electrons in SnO_2, and $2.76 \times 10^{18}\ cm^{-3}$ for holes in NiO. Based on the potential-related depletion length equation below, the depletion length for NiO and SnO_2 are summarized in **Supplementary Table 3**. x_p and x_n are the negative and positive depletion layer widths. At the same time, N_D and N_A are the carrier concentration of donors and acceptors. q is the electron charge, and V_d is the built-in potential.

$$x_p = \left[\frac{2\varepsilon V_b}{q} \frac{N_D}{N_A(N_A + N_D)}\right]^{1/2}$$

$$x_n = \left[\frac{2\varepsilon V_b}{q} \frac{N_A}{N_D(N_A + N_D)}\right]^{1/2}$$

Supplementary Table 3 Carrier concentration and depletion region analyzed by Hall effect measurement.

	**Carrier concentration**	x_p **or** x_n	**Depletion region width**
P-type	$+2.76 \times 10^{18}\ cm^{-3}$	$16.7\ nm$	$\sim 29.8\ nm$
N-type	$-3.20 \times 10^{18} cm^{-3}$	$13.1\ nm$	

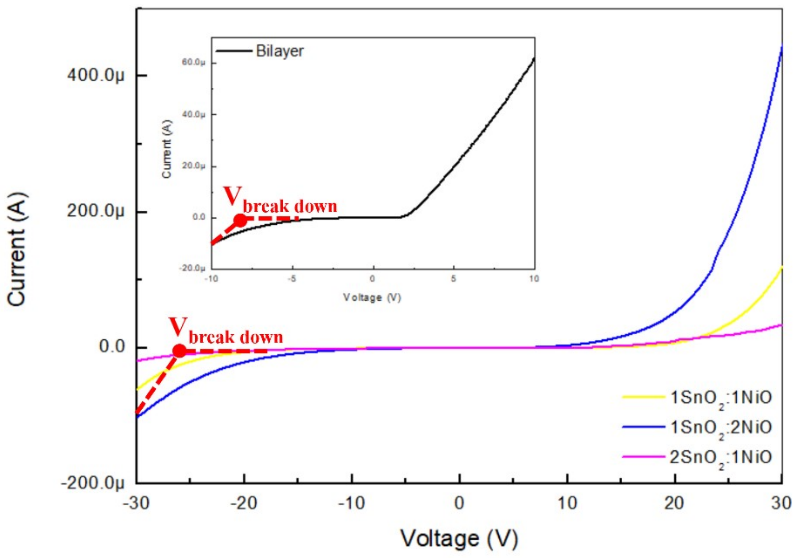

Supplementary Fig. 12 Current-voltage characteristic of bilayer SnO_2/NiO, SnO_2:NiO with composition of 0.33, 0.5, 0.67 of Ni.

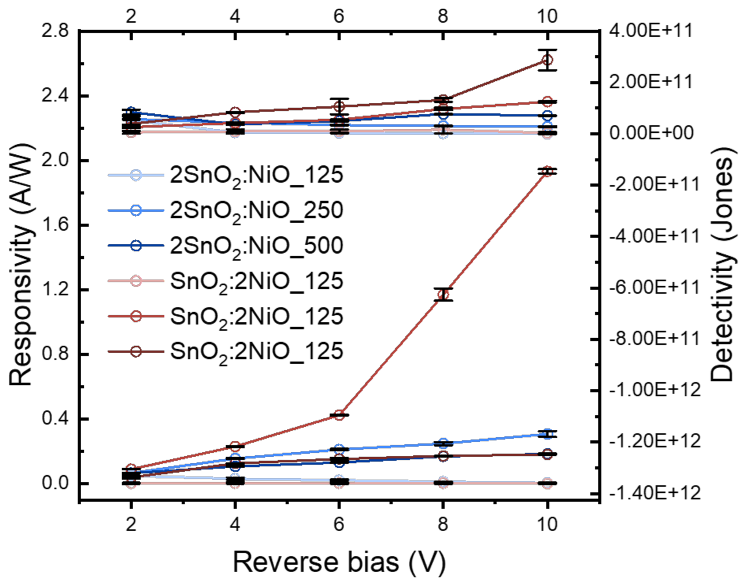

5

Supplementary Fig. 13 The photovoltaic response of annealed SnO_2:NiO with the composition ratio of 2:1 (pillar) and 1:2 (bi-continuous) with various thicknesses of 125 nm, 250 nm, and 500 nm.

The photoconduction with the composition ratio of 2:1 (pillar) and 1:2 (bi-continuous) samples with various thicknesses (125 nm, 250 nm, and 500 nm). The result is shown in supplementary Fig. 13, and the optimum thickness for responsivity is determined to be 250 nm for a 1:2 (bi-continuous) sample. In addition, higher detectivity is found at the thickness of 500 nm in a 1:2 (bi-continuous) sample, and the same tendency is found for the 2:1 (pillar) sample. According to the equations for responsivity and detectivity below, the responsivity is proportional to the difference between leakage current and photocurrent. In the meantime, the detectivity further depends on the value of the dark current.

$$\mathrm{R}=\frac{J_{\mathrm{light}}-J_{dark}}{\mathrm{P}_{in}}, \quad \mathrm{D}^{*}\cong\frac{\mathrm{R}}{\sqrt{2qJ_{dark}}}$$

J_{light}, J_{dark}, and P refer to the light current density, dark current density, and light intensity, respectively. According to the leakage current (dark current) and photocurrent illustrated in Supplementary Table 4, the leakage current is reduced with a higher thickness of 500 nm. Therefore, although the photocurrent (difference between dark and light current) is reduced at 500 nm, higher detectivity is expected.

Supplementary Table 4 The dark current (leakage current), photocurrent, and responsibility of annealed SnO_2:NiO with the composition ratio of 2:1 (pillar) and 1:2 (bi-continuous) with various thicknesses of 125 nm, 250 nm, and 500 nm.

	Dark current at 10 V (A)	Photocurrent at 10V (A)	Responsivity (A/W)
(2:1) pillar 125 nm	7.08E-08	7.17E-08	7.64E-03
(2:1) pillar 250 nm	1.13E-07	1.52E-07	3.34E-01

(2:1) pillar 500 nm	6.88E-09	2.85E-08	1.83E-01
(1:2) bi-continuous 125 nm	1.60E-10	1.83E-10	1.93E-04
(1:2) bi-continuous 250 nm	2.24E-07	4.53E-07	1.95E+00
(1:2) bi-continuous 500 nm	3.85E-10	2.19E-08	1.82E-01

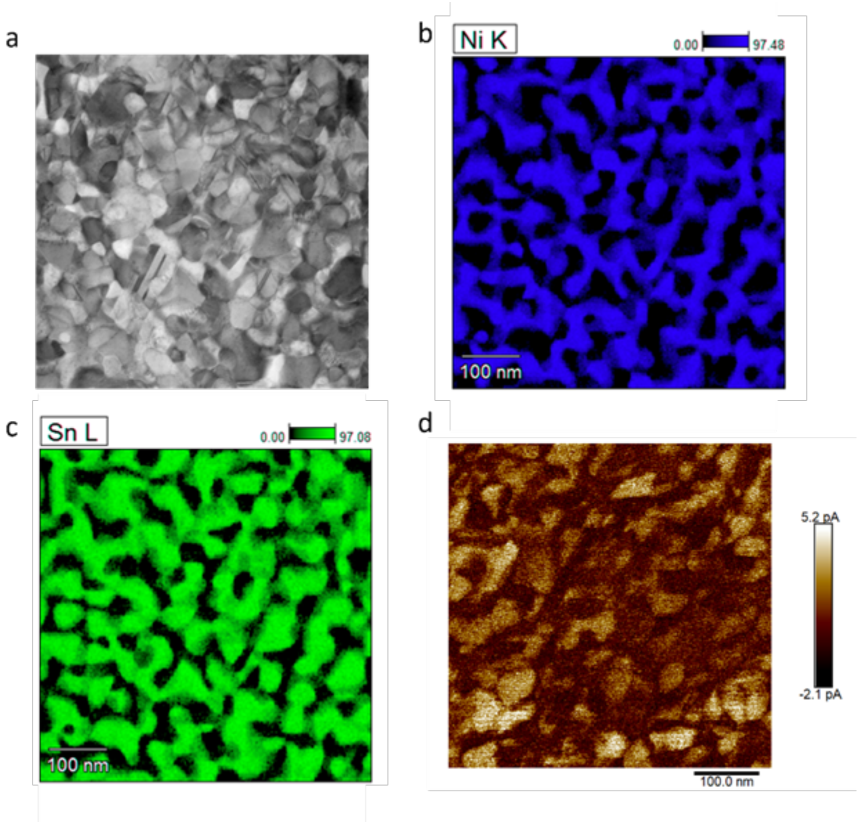

Supplementary Fig. 14 Comparison between STEM-EDS and photo-current.

1. Zhu J, Chen L-Q, Shen J, Tikare V. Coarsening kinetics from a variable-mobility Cahn-Hilliard equation: Application of a semi-implicit Fourier spectral method. *Physical Review E* **60**, 3564-3572 (1999).

2. Brunini VE, Schuh CA, Carter WC. Percolation of diffusionally evolved two-phase systems. *Physical Review E* **83**, 021119 (2011).

3. Hu J-M*, et al.* Phase-Field Based Multiscale Modeling of Heterogeneous Solid Electrolytes: Applications to Nanoporous Li_3PS_4. *ACS Appl Mater Interfaces* **9**, 33341-33350 (2017).

4. Chen L-Q, Zhao Y. From classical thermodynamics to phase-field method. *Progress in Materials Science* **124**, 100868 (2022).

5. Sheng G, Wang T, Du Q, Wang K, Liu Z, Chen L. Coarsening kinetics of a two phase mixture with highly disparate diffusion mobility. *Commun Comput Phys* **8**, 249-264 (2010).

6. Chen LQ, Shen J. Applications of semi-implicit Fourier-spectral method to phase field equations. *Comput Phys Commun* **108**, 147-158 (1998).

7. Echeverría-Alar S, Clerc MG. Labyrinthine patterns transitions. *Physical Review Research* **2**, 042036 (2020).

8. Yamanouchi M, Jander A, Dhagat P, Ikeda S, Matsukura F, Ohno H. Domain Structure in CoFeB Thin Films With Perpendicular Magnetic Anisotropy. *IEEE Magnetics Letters* **2**, 3000304-3000304 (2011).
